# Bergamot (*Citrus bergamia*, Risso): The Effects of Cultivar and Harvest Date on Functional Properties of Juice and Cloudy Juice

**DOI:** 10.3390/antiox8070221

**Published:** 2019-07-12

**Authors:** Angelo Maria Giuffrè

**Affiliations:** Università degli Studi Mediterranea di Reggio Calabria, AGRARIA—Dipartimento di Agricoltura, Risorse forestali, Ambiente Risorse zootecniche, Ingegneria agraria, Alimenti—Contrada Melissari, 89124 Reggio Calabria, Italy; amgiuffre@unirc.it

**Keywords:** antioxidants, bergamot, bioactive compound, biometrics, biomolecules, *Citrus bergamia* Risso, cloudy juice, non-climacteric

## Abstract

Reggio Calabria province (South Italy) is known for being almost the only area of cultivation of the bergamot fruit, grown principally for its essential oil, but today much studied for the health benefits of its juice. The biometrics and physico-chemical properties of the three (*Citrus bergamia* Risso) existing genotypes namely Castagnaro, Fantastico and Femminello were studied during fruit ripening from October to March. Castagnaro cultivar had the biggest and heaviest fruit during this harvest period. °Brix (7.9–10.0), pH (2.2–2.8) and formol number (1.47–2.37 mL NaOH 0.1 N/100 mL) were shown to be influenced by both the genotype and harvest date. Titratable acidity (34.98–59.50 g/L) and vitamin C (ascorbic acid) (341–867 g/L) decreased during fruit ripening. The evolution of flavonoids such as neoeriocitrin, naringin, neohesperidin, brutieridin and melitidin was studied both in bergamot juice and in the bergamot cloudy juice which is the aqueous extract of bergamot during fruit processing. Bergamot cloudy juice contained a higher quantity of flavonoids compared to the juice. This study gives important information regarding the cultivar and the harvest date for producers who want to obtain the highest juice quantity or the highest juice quality from the bergamot fruit.

## 1. Introduction

Bergamot (*Citrus bergamia*, Risso) is an evergreen tree almost exclusively grown on the Ionian and Tyrrhenian Coast of Reggio Calabria Province (South Italy), in a strip of land 1–12 km wide. Given its economic benefits, bergamot is very important to the region where it is cultivated [1]. Three cultivars (cv) (genotypes) are known: Castagnaro, Fantastico and Femminello. In 2017, 1500 hectares of bergamot trees were cultivated in Reggio Calabria, producing 18,750 tons of fruits [2]. Bergamot is a non-climacteric fruit [3] and in the past was picked when the highest essential oil content in the peel was reached. Today the juice is also considered. The fruit was commonly cultivated for its essential oil extracted from the peel, which is used in the cosmetic, perfumery [4] and food industries [5]. Very recently the bioconversion of juice and peel into wines and vinegars was positively conducted [6]. More recently there has been an increasing interest in the use of its juice as a beverage and also in a blend with other fruit juices. This interest is related to the demand for minimally processed foods and functional foods containing antioxidants and biomolecules whose beneficial effects on human health have been widely studied regarding diabetes, cancer, Alzheimer’s disease, insulin resistance and neuro-disease [7]. There is no universally accepted definition of functional food. The following definition could be applied: “Natural or processed foods that contain known or unknown biologically-active compounds; which in defined amounts provide a clinically proven and documented health benefit for the prevention, management, or treatment of chronic disease” [8]. This merges with, and updates, the definitions stated by the National Academy of Sciences Food and Nutrition Board in the United States [9], the Institute of Food Technologists [10], the American Dietetic Association [11] and what De Felice stated for nutraceuticals [12]. The aim of this research was to investigate the evolution of biometrics and the anti-oxidative properties during the fruit ripening (six months) to evaluate the right harvest date to obtain the highest physico-chemical quality of bergamot, bergamot juice and bergamot cloudy juice. In this context, the effect of cultivar was also studied.

## 2. Materials and Methods

### 2.1. Plant Material

All three cultivars were grown in the same area, in mono-cultivar plots. The bergamot trees were cultivated by experienced growers on level ground and were planted with a distance of 6 m between each row and the trees were 5 m apart within each row. All the trees (25–30 years old) were irrigated and fertilized in the same way. Fertilization was conducted by using 5 kg per tree of a complex fertilizer (nitrogen, phosphorus and potassium, NPK, 20:10:10) in multiple rates from late winter to early spring and calcium nitrate 200 g/tree/month from July to September. A drip irrigation was conducted from March to late October in relation with the environmental temperature and based on the demand for water by trees (evapotranspiration). The soil was slightly acidic, deep and well drained because bergamot roots suffer water stagnation. Fruits were collected early in the morning and carefully placed in plastic containers commonly used for citrus fruit picking. Thereafter, fruits were immediately transferred to the laboratory for biometric analyses. Thirty kilograms of bergamot fruits were randomly collected from 20 trees at each harvest date for each cultivar (Castagnaro, Fantastico and Femminello) in the middle of each month from October 2016 to March 2017. For each cultivar, two batches (15 kg each) were prepared at each harvest date and two replicates were obtained from each batch. The same experimental design was replicated in the harvest year 2017–2018.

### 2.2. Juice Extraction

The bergamot fruit is cultivated for essential oil extraction (from peel) and juice extraction. In the present study both the ‘albedo’ and the remaining pulp which are commonly known as ‘pastazzo’ were processed into hammer mills where they were grinded and homogenized with water to solubilize polyphenols. The obtained mixture undergoes various steps in steel tanks to allow the flavonoids to be extracted as much as possible from the ‘pastazzo’ before transferring to the liquid phase. A first rough separation is conducted by a press, which divides the pulp destined for the subsequent recovery of the pectins in another production plant, from the liquid phase which is stored in steel tanks for subsequent processing. The pressed pulp contains a large quantity of pectin, both water soluble and nonsoluble, which makes the separation of pulp from the liquid fraction difficult. For this reason, pectinase was used as a pectolytic enzyme at 50–60 °C to facilitate pectin degradation. The juice obtained by this procedure is called ‘cloudy juice’. Pectinase is commonly used to break up the cell wall and to intensify the phenolic compounds extraction [13,14,15]. The extraction method applied in this work is commonly used for edible juice extraction.

### 2.3. Chemicals

Chemicals of both analytical grade and chromatographic grade were purchased from Carlo Erba, Milan, Italy. 2,2-Diphenyl-1-picrylhydrazyl (DPPH) and pure standards of naringin, neoeriocitrin and neohesperidin, were from Sigma-Aldrich Chemical Co. (St. Louis, MO, USA). TPTZ (2,4,6-tripyridyl-s-triazine for FRAP reagent (Ferric Reducing Ability of Plasma) was from Fluka Chemicals Switzerland. Brutieridin and melitidin as pure standards were obtained as described by Di Donna et al. [16].

### 2.4. Pulp Content

Pulp content is the solid fraction quantified as a percentage in bergamot juice after centrifugation for 10 min at 3500 rpm. Pulp and juice are separated by difference of gravity.

### 2.5. Turbidity

A 12% bergamot juice in bi-deionized water was prepared, and transmittance was read at 578 nm. Turbidity was expressed as a percentage ratio between the intensity of the incident light and intensity of light emission from the cuvette.

### 2.6. pH

A Mettler Toledo instrument was used after calibration pH 7.0 and pH 4.0.

### 2.7. °Brix

The degree Brix was determined by a Mettler Toledo refractometer on a drop of bergamot juice sample after zero-set of the instrument by a drop of bi-deionized water.

### 2.8. Titratable Acidity

A 10 g aliquot of bergamot juice and 150 mL of bi-deionized water were placed in a glass beaker. The mixture was boiled for 10 min. Thereafter acidity was determined by titration with a 0.1 NaOH aqueous solution up to pH 8.1. Acidity was expressed as g of citric acid monohydrate per liter of juice [17].

### 2.9. Vitamin C

Vitamin C was quantified by an iodomeric titration. In a glass beaker, 1 mL of bergamot juice and 5 mL of bi-deionized water were mixed and titrated by a 0.01 N iodine solution using a 2% starch solution as an indicator. The result was expressed as mg ascorbic acid/L of juice [17].

### 2.10. Formol Number

In a glass beaker, 10 mL of bergamot juice, 10 mL of 40% by volume formaldehyde solution (pH 2.8) and 7 drops of phenolphthalein (1% in ethanol) were measured. The mixture was stirred and titrated by a 0.1 NaOH solution (IFUMA 30, method EN 1133) [18].

### 2.11. Flavonoids

The analysis was carried out using the method suggested by Giuffrè et al. [6] and modified using a HPLC-PDA system (i.e. a liquid chromatograph coupled with a photo diode array detector) and equipped with a column conditioning system at 25 °C. The separation column was a Kinetex 5μ C18 100 Å, 150 mm length, 4.6 mm internal diameter. The mobile phase was 0.1% formic acid in deionized water (A) and methanol (B) with the following conditions: 80% A in isocratic (5 min); from 80% A to 45% A in gradient (42 min); 45% A in isocratic (5 min); from 45 to 20% A (7 min); 20% A in isocratic (5 min); from 20 to 80% A (5 min). The injection volume was 20 μL and the flow rate was set at 1 mL/min. The system was supported by Chromera software version 3.4.0.5712.

The limit of detection and linearity of the detector response were determined by a five points calibration curve of flavonoids. Triplicate standards solutions of neoeriocitrin, naringin and neohesperidin were prepared at 5, 10, 25, 50 and 100 mg/100 mL in methanol, from a solution of 250 mg/100 mL.

### 2.12. DPPH and FRAP Assays

The analyses were conducted spectrophotometrically as suggested by Panuccio et al. and Sicari et al. [19,20].

### 2.13. Statistical Analysis

Means and standard deviations were calculated on 8 replicates (4 replicates × 2 harvest years) by Excel 2010 software. Statistical differences were calculated by one-way ANOVA and Tukey test for post hoc analysis at *p* < 0.05 using the SPSS 17.0 software (SPSS Inc., Chicago, IL, USA); the variables were: the cultivar and the harvest date of bergamots. Principal component analysis (PCA) was carried out using the software XLSTAT version 2009.1.01.

## 3. Results and Discussion

### 3.1. Biometrics

The vertical diameter length was longest in the Castagnaro fruit which showed the most constant increase in length, from 6.63 cm in October to more than 9 cm after December. The vertical diameter length of Fantastico and Femminello fruits showed a slight decrease in March at the end of the ripening period (7.15 and 6.50 cm, respectively) (Table 1). The horizontal diameter was greatest in Castagnaro at each monthly sampling and showed a tendency to increase during the ripening of Castagnaro and Fantastico, whereas in Femminello a slightly fluctuating rate was found (Table 1). The pulp in juice content showed very high significant differences (*p* < 0.001) for each cultivar during fruit ripening but no significant differences were found between cultivars in October, December and January (Table 1). Vertical diameter increased with horizontal diameter (r = 0.958), fruit weight (r = 0.87) and peel weight (r = 0.880), (Table 2). The increase in fruit weight was highly significant (*p* < 0.001) during fruit ripening of all cultivars. From October to March, the Castagnaro fruit showed both the highest increase in weight during ripening (72%) and the highest weight each month (245 g in October and 421 g in March). Fantastico fruits increased by 49% during ripening (173 g in October and 258 g in March). Femminello fruits showed both the lowest weight on each harvest date and the lowest increase in weight (23%) from October to February (Table 3). The peel weight was always greatest in Castagnaro (44.1 g in October, 55.01 g in December and 74.02 g in February), but a substantial fall in weight was measured in March in all three cultivars (Table 3). The percentage of juice was highest in Fantastico at the first stage of ripening (29.33%–30.50%) and in Fantastico and Femminello at the end of the ripening period (39.97% and 40.01%, respectively) (Table 1). Pulp in juice is a negative parameter because it has to be removed during the industrial process before using or storing the fruit juice. The juice turbidity was very highly significantly different (*p* < 0.001) during ripening and the same significance of differences was found between cultivars at each monthly sampling (Table 3). In the correlation matrix, fruit weight had a strong positive correlation with the vertical diameter (r = 0.875; *p* < 0.001; r^2^ = 0.766; t = 19.33) and a stronger correlation with the horizontal diameter (r = 0.920; *p* < 0.001; r^2^ = 0.846; t = 19.25) (Table 2). The increase in peel weight in the three cultivars was strongly correlated with the vertical diameter (r = 0.880; *p* < 0.001; r^2^ = 0.774; t = 14.76) and with the horizontal diameter (r = 0.830; *p* < 0.001; r^2^ = 0.689; t = 14.62) (Table 2). Fruit weight showed a weak correlation with juice content (r = 0.112; *p* < 0.001; r^2^ = 0.013; t = 17.23) but it was strongly correlated with peel weight (r = 0.815; *p* < 0.001; r^2^ = 0.664; t = 16.56). This means that the increase in weight during fruit ripening is mainly due to the increase in peel and not that of pulp.

### 3.2. pH

Bergamot juice is very acidic and mainly contains ascorbic and citric acid which contribute significantly to the composition of this parameter. Between cultivars, no significant differences were found in November, February and March. A very high significant pH increase (*p* < 0.001) was found in Castagnaro and Femminello fruit juices and high significant differences were found in Fantastico juice (*p* < 0.01) (Table 4). pH of juice was negatively and moderately correlated with vitamin C (r = 0.643; *p* < 0.001; r^2^ = 0.413; t = 31.32), but strongly and negatively correlated with titratable acidity (r = 0.740; *p* < 0.001; r^2^ = 0.548; t = 50.76) (Table 5). The pH of the bergamot juice was lower than grapefruit juice (3.05), orange juice (3.63) and tangerine juice (3.41) but similar or higher than lemon juice (2.43) [21].

### 3.3. °Brix

The degree Brix is the sugar content expressed as g/100 g juice. It is directly proportional to the sweetness of the fruit and therefore to its organoleptic pleasantness. This value did not exceed 10, which was reached by Fantastico cv in November (Table 4). The analysis of variance showed very highly significant differences during ripening (*p* < 0.001) in all the cultivars. If the cultivar effect is considered, very high significant differences in November, January and March (*p* < 0.001) were found, high significant differences in October and December (*p* < 0.01) and significant differences (*p* < 0.05) in February (Table 4). The °Brix/titratable acidity (%) is a maturity index and the highest value for all the three cultivars was found in the last month of sampling, with a tendency to increase during ripening (Table 4). The °Brix/titratable Acidity (%) ratio had a strong negative correlation with total flavonoids in juice (r = 0.920; *p* < 0.001; r^2^ = 0.846; t = 16.61) (Table 5). Other Authors found different °Brix in other citrus juices: 5.10 (grapefruit), 1.16 (lemon), 4.53 (orange), 6.50 Tangerine [21], and 11.0 °Brix in squeezed blood orange juice cultivated in Calabria [22].

### 3.4. Titratable Acidity

The titratable acidity is an important parameter to determine the maturity of the fruit and the acidic taste in citrus fruit. The degree of maturity of a fruit is one of the most important factors to determine conservation methods and control quality parameters such as taste and flavor. An immature fruit usually has a low sugar content in relation to acidity, compared to a ripe fruit that has a high level of sugar in relation to acidity. In bergamot juice a very high significant difference in titratable acidity between cultivars (*p* < 0.001) was observed, from a low 53.86 g/L in Castagnaro to a high 58.67 g/L in Fantastico, measured at the earliest sampling event (Table 4). During fruit ripening a decreasing trend in titratable acidity in all cultivars was observed. At the last sampling event in Castagnaro the lowest content (34.98 g/L) was seen, namely a decrease of 35.05% from October to March, whereas the highest value was not found in Fantastico (as at the earliest sampling event) but in Femminello (41.90 g/L) with a decrease rate of 22.81%.

### 3.5. Vitamin C

The human body cannot synthesize vitamins; therefore, they have to be part of our diet. Vitamin C (ascorbic acid) is water soluble, has antioxidant potential [23], prevents scurvy [24] and degenerative diseases, particularly those that are ageing-related [25] and has possible protective effects on the bones of older adults. Vitamin C can be oxidized by storage at room temperature, the addition of baking soda, overcooking, contact with copper and over intakes of zinc (cooking tools), alcohol and pectin [26]. In the studied samples, vitamin C decreased dramatically during fruit ripening: Castagnaro (59%), Fantastico (47%) and Femminello (48%) from October to March. In October a very high significant difference in vitamin C between cultivars (*p* < 0.001) was found: 831 mg/L in Castagnaro, 867 mg/L in Fantastico and 669 mg/L in Femminello (Table 4). Vitamin C content was not influenced by cultivars in February but was very highly affected by this variable (*p* < 0.001) in all other months (Table 4). Findings of other Authors on vitamin C content in citrus fruit juices revealed 680 mg/L and 455 mg/L, respectively in Marsh and Star Ruby (i.e., two grapefruit cultivars [27]), 680 mg/L in blood orange [17], 220 mg/L in pomelo [28] and 355 mg/L in lemon analyzed by HPLC [21].

### 3.6. Formol Number

The Formol number can represent, in a normal chemical industrial control, a useful index for the global quantitative evaluation of amino acids present in fruit juices. The Formol number is not influenced by the presence of many natural constituents of fruit juice (sugars, vitamins, flavorings, colorings) and it is applied in the quality determination of fruit juice and beverages because it expresses the total number of amino acids found. In bergamot juice the cultivar did not influence the Formol number in October but very high significant differences (*p* < 0.001) were found between cultivars from November to March. The harvest date had significant influence (*p* < 0.001) on the Formol number for the three cultivars (Table 4). The Formol number varied from 23.7 mL NaOH 0.1 N/100 mL (Femminello in March) to 14.3 mL NaOH 0.1 N/100 mL (Castagnaro in December and Femminello in February) and exceeded 20 mL NaOH 0.1 N/100 mL at the same time in all the three cultivars only in October. No correlation was found between Formol number and melitidin in juice (r = 0) (Table 5).

### 3.7. Flavonoids

Flavonoids are polyphenols with an antioxidant and radical scavenging role and are described by the scientific literature to have many beneficial effects on human health. They are biomolecules that prevent the risk of primary open-angle glaucoma [28], have an antiplatelet effect [29], maintain the anti-inflammatory action of cortisol under pro-oxidant conditions [30], protect vascular endothelial function [31], have an anti-obesity activity [32], reduce the risk of cardiovascular disease [33] and have antimicrobial [34], antiviral [35] and anti-inflammatory effects [36]. Neoeriocitrin was significantly high in Femminello and low in Castagnaro and Fantastico (*p* < 0.001) at the earliest sampling event. This compound showed a tendency to increase in the bergamot juice of Castagnaro and Fantastico as the fruits ripened from October to February, with a fall in March (Table 6). Naringin, neohesperidin and brutieridin were the major flavonoids in the bergamot juice (Table 6), whereas neoeriocitrin, naringin and neohesperidin prevailed in bergamot cloudy juice (Table 7); this was probably due to a higher water solubility. Naringin was very highly significantly influenced (*p* < 0.001) by both cultivar and harvest date variables (Table 6 and Table 7). Neoeriocitrin in both bergamot juice and bergamot cloudy juice was highest in the last fruit sample dates (February and March) for all three cultivars (Table 6 and Table 7). In the bergamot juice, naringin content was highest on the last sample date (42.61%, 28.63% and 42.30% of the total flavonoids, respectively, for Castagnaro, Fantastico and Femminello). Neohesperidin in bergamot juice was significantly different at each sample date (*p* < 0.001) with January being the month in which the highest quantity was measured. Almost always, in both the juice and the cloudy juice of the three cvs of bergamot, neohesperidin content was highest in Fantastico (Table 6 and Table 7). Brutieridin and melitidin are two molecules identified and described in bergamot juice by Di Donna et al. [16,37,38,39] and Fiorillo et al. [40]. The name brutieridin comes from the ancient name of one of the Calabrian cities (Brutium, today Cosenza) where brutieridin and melitidin were studied, whereas melitidin derives from the name of one of the most important towns (Melito Porto Salvo) where the bergamot tree is cultivated. In the samples studied in our work, brutieridin was always greater than melitidin, both in bergamot juice and in bergamot cloudy juice (Table 6 and Table 7). On the first sample dates (October–December), melitidin was higher in Castagnaro and Femminello juice than in Fantastico, and the same situation was found on the last sample date (Table 6). Melitidin in cloudy juice was significantly lower in March when its content was lower than 3% in all the three cultivars, whereas it was double or almost double in the early period of ripening from October to December (Table 7). A significant decreasing tendency in the brutieridin content of bergamot juice was recorded in Castagnaro and Fantastico (*p* < 0.001), whereas a fluctuating rate was found during fruit ripening in Femminello juice and in the cloudy juice of the three cultivars. Brutieridin detected in both bergamot juice and bergamot cloudy was almost always greatest in Fantastico cv from October to March, except in February when the highest brutieridin content was found in Femminello (26.94%) for bergamot juice and in Castagnaro (14.33%) for bergamot cloudy juice. Harvest date and cultivar variables showed very high significant differences (*p* < 0.001) between means (Table 6 and Table 7). The correlation between each single flavonoid in juice and its homologous in cloudy juice was between r = 0.217 of brutieridin and r = 0.582 of melitidin (Table 5). In the bergamot juice the total flavonoid content constantly increased with fruit ripening in Fantastico from 361 mg/L in October to 678 mg/L in March (namely an increase rate of 87.81%), and in Femminello from 287 mg/L in October to 824 mg/L in March (namely an increase of 187.11%). Also, in Castagnaro juice the flavonoid content was higher in the last period of fruit ripening compared to October and November (Table 6). Studies on flavonoid content in other citrus juice during storage at 4 °C showed a decreasing trend [41], which indicate fruit should be picked later, and juice should be consumed as soon as possible after picking.

### 3.8. DPPH Assay and FRAP Assay

A citrus juice contains more than one class of antioxidants which have different behaviors. For this reason, many authors suggest applying more than one assay to evaluate antioxidant activity. In the present study we applied DPPH assay and FRAP assay which are two of the most common tests used on many matrices such as the common orange [20], blood orange juice [17], edible vegetable oils and potential industrial vegetable oils [42,43,44], apples, bananas, strawberries, kiwifruit and cauliflower [45]. Vitamin C and flavonoids are the most important antioxidants in bergamot juice and show an inverse ratio during fruit ripening: the vitamin C showed a decreasing trend (Table 4) in opposition to total flavonoid content which increased with harvest date (Table 6). In all the three cultivars DPPH value showed a very high significant difference at each month of sampling (Table 8). The correlation between antioxidant activity of the bergamot juice measured with the DPPH assay was high with total flavonoid content (r = 0.764; *p* < 0.001; r^2^ = 0.583; t = 11.87). This was in accordance with results of Roussos [46] which found a strong positive correlation between DPPH and flavonoids in blood orange juice. FRAP assay had an almost strong positive correlation with the titratable acidity (r = 0.673; *p* < 0.001; r^2^ = 0.453; t = 29.45) and with brutieridin in juice (r = 0.699; *p* < 0.001; r^2^ = 0.488; t = 9.73), and a moderate positive correlation with °Brix (r = 0.441; *p* < 0.001; r^2^ = 0.194; t = 41.07) and neohesperidin in cloudy juice (r = 0.385; *p* < 0.001; r^2^ = 0.148; t = 8.97). FRAP assay was also moderately correlated with melitidin in both juice (r = 0.402; *p* < 0.001; r^2^ = 0.161; t = 35.96) and cloudy juice (r = 0.406; *p* < 0.001; r^2^ = 0.165; t = 44.75) showing a very similar Pearson coefficient (Table 8). Lastly, vitamin C was found to be responsible for the antioxidant activity measured by FRAP assay (r = 0.962; *p* < 0.001; r^2^ = 0.926; t = 29.45) (Table 5), similar to the findings of other authors on citrus juices [47,48,49], but in partial disagreement with other results [50].

### 3.9. Hierarchical Cluster Analysis and Principal Component Analysis

**Figure 1:**
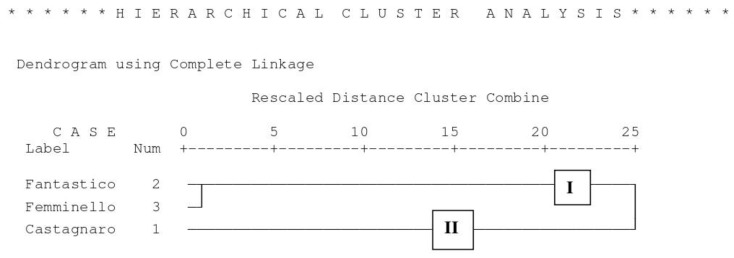
Two-dimensional dendrogram obtained from the cluster analysis of the fruit biometrics and of the physico-chemical properties of juice and cloudy juice of the bergamot fruit (*Citrus bergamia*, Risso).

**Figure 2:**
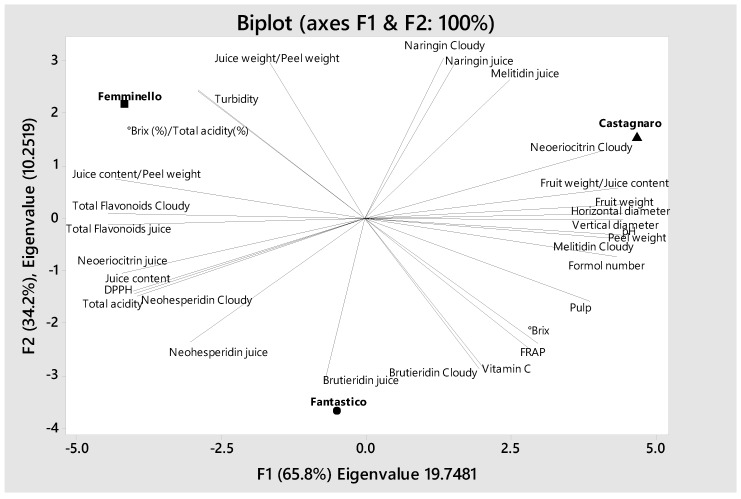
Score plot of the principal component analysis (PCA) performed on the biometrics and the physico-chemical properties of juice and cloudy juice of the three cultivars (Castagnaro, Fantastico and Femminello) of the bergamot fruit (*Citrus bergamia*, Risso).

The three cultivars were found to cluster into two clades (Figure 1). Clade 1 contained Fantastico and Femminello which showed a high similarity and were clustered at a distance of 1. The second cluster contained the Castagnaro cv alone, with the highest fruit and peel weights; in particular, Castagnaro showed a peel weight double or more than double that of Femminello. In Castagnaro the highest vertical and horizontal diameters were also found, as well as the lowest flavonoid content in juice and cloudy juice and the lowest titratable acidity. Principal component analysis (PCA) was performed on the three cultivars and 30 parameters were included in the test (Figure 2). Two Eigen values were obtained and together accounted for 100% of the cumulative variability. The Eigen values and the percentage of total variance were 19.7481 (65.8%) and 10.2519 (34.2%). The visualization of the discrimination between the different orange cultivars on the plane of the first two functions led to a distinct separation. The cultivars were split between three sides of the plane which demonstrate the significant difference among the cultivars. The graphic also shows how the parameters are linked or separated from the cultivar factor. The Castagnaro cultivar located in the right corner of the plane is linked to the neoeriocitrin cloudy juice and fruit weight/juice content ratio. The Fantastico cultivar located in the lower center of the plane, is more influenced by the brutieridin in juice and in cloudy juice. Finally, the Femminello cultivar, which is located in the left corner of the plane, is influenced by the °Brix/titratable acidity (%) ratio, turbidity and juice content/peel weight ratio. Some parameters showed an independency from the cultivar factor because of the their location in the plane, such us the naringin in cloudy juice, the naringin in juice, °Brix, FRAP value, DPPH value, titratable acidity, neohesperidin and total flavonoids in juice and cloudy juice. Also, some parameters are correlated negatively, such as neohesperidin and naringin, as these parameters are located in opposite directions in the plane.

## 4. Conclusions

Bergamot is a tree and fruit with a very strong geographical connotation, growing almost exclusively in Reggio Calabria province (South Italy). Three cultivars of this citrus genus are known (Castagnaro, Fantastico and Femminello) and this study has shown a strong effect of both the cultivar (genotype) and the harvest date. These variables were found to influence the biometrics and the physico-chemical properties of fruits and fruit juice. Castagnaro is the cultivar producing the heaviest fruit, with the highest vertical and horizontal diameter and with the highest peel content during fruit ripening from October to March. Vitamin C content decreases during bergamot fruit ripening and it is close to the mean or in a higher quantity compared to other citrus fruit juice. The findings of this study show that the bergamot fruit is a very good source of flavonoids which can be directly used in food and beverage preparation when obtained from fruit juice and pulp, or which can be extracted from cloudy juice for food, beverages and pharmaceutical purposes. Naringin and neohesperidin were two flavonoids predominating in both the bergamot juice and in the bergamot cloudy juice with brutieridin as one of the two most represented flavonoids in bergamot juice and naringin as the most represented flavonoid in the bergamot cloudy juice. Brutieridin and melitidin are two flavonoids characterizing bergamot juice.

## Figures and Tables

**Table 1:** Biometrics of bergamot fruit. Results are presented as the mean value ± standard deviation, n = 8, (2016–2017 and 2017–2018 harvest years).

	Cultivar	October	November	December	January	February	March	Sign.
**Vertical** **Diameter (cm)**	Castagnaro	8.63 ± 0.25 cA	8.67 ± 0.10 bcA	9.02 ± 0.10 abA	9.07 ± 0.06 aA	9.04 ± 0.09 aA	9.0 ± 0.10 abA	**
	Fantastico	7.33 ± 0.06 cB	7.3 ± 0.10 cB	8.12 ± 0.10 aB	8.11 ± 0.09 aB	7.65 ± 0.09 bB	7.15 ± 0.09 cB	***
	Femminello	6.90 ± 0.1 aC	6.53 ± 0.25 abC	6.47 ± 0.15 bC	6.83 ± 0.06 abC	6.83 ± 0.06 abC	6.5 ± 0.17 abC	**
	Sign.	***	***	***	***	***	***	
**Horizontal Diameter (cm)**	Castagnaro	8.8 ± 0.10 bA	8.7 ± 0.20 bA	9.77 ± 0.15 aA	9.60 ± 0.10 aA	9.43 ± 0.03 aA	9.77 ± 0.12 aA	***
	Fantastico	7.13 ± 0.06 cB	7.2 ± 0.10 cB	8.40 ± 0.05 aB	8.36 ± 0.06 aB	7.98 ± 0.08 bB	8.04 ± 0.06 bB	*
	Femminello	7.07 ± 0.25 aB	6.53 ± 0.21 bC	6.67 ± 0.12 a bC	7.1 ± 0.10 aC	7.03 ± 0.25 abC	6.77 ± 0.15 abC	*
	Sign.	**	***	***	***	***	***	
**Pulp in Juice** **(%)**	Castagnaro	10.17 ± 0.29 bA	10.00 ± 0.50 bB	7.00 ± 0.50 cA	9.83 ± 0.29 bA	10.33 ± 0.58 bA	11.67 ± 0.58 aAB	***
	Fantastico	10.33 ± 0.58 aA	10.33 ± 0.58 aA	7.33 ± 0.58 bA	10.33 ± 0.58 aA	10.33 ± 0.58 aA	10.00 ± 1.0 aB	***
	Femminello	10.00 ± 0.0 bA	9.00 ± 0.0 cB	8.00 ± 0 dA	10.00 ± 0.0 bA	7.03 ± 0.06 eB	12.17 ± 0.29 aA	***
	Sign.	n.s.	*	n.s.	n.s.	***	**	

*** significance at *p <* 0.001; ** significance at *p <* 0.01; * significance at *p <* 0.05; n.s., not significant. Means in the same line are distinguished by small letters. Means in the same column are distinguished by capital letters.

**Table 2:** The correlation matrix of biometrics is built on the basis of 48 values for each parameter (4 replicates × 2 harvest years × 3 cultivars). In the south-west section of the matrix is the r-value (above) and the significance level (below) with *p* < 0.001,***; n.s., not significant. In the north-east section of the matrix is the *t*-value (in italics) with the significance of the *t*-test calculated at 95% confidence interval and the R^2^ value (underlined).

	Vertical Diameter	Horizontal Diameter	Pulp in Juice	Fruit Weight	Peel Weight	Juice Content	Fruit Weight/Peel Weight	Fruit Weight/Juice Content	Juice Content/Peel Weight	Turbidity
**Vertical Diameter**	****1****	*1.38* 0.918	*7.99* 0.002	*19.33* 0.766	*14.76* 0.774	*26.50* 0.013	*4.59* 0.028	*0.28* 0.702	*45.50* 0.587	*37.96* 0.000
**Horizontal Diameter**	0.958 n.s.	**1**	*6.50* 0.001	*19.25* 0.846	*14.62* 0.689	*26.10* 0.002	*5.47* 0.000	*0.37* 0.638	*41.89* 0.426	*37.44* 0.009
**Pulp in Juice**	0.048 ***	0.024 ***	**1**	*19.12* 0.001	*13.78* 0.000	*24.05* 0.000	*10.60* 0.031	*3.98* 0.003	*40.54* 0.001	*34.96* 0.034
**Fruit Weight**	0.875 ***	0.920 ***	0.090 ***	**1**	*16.56* 0.664	*17.23* 0.013	*19.37* 0.034	*19.26* 0.619	*19.84* 0.345	*16.69* 0.000
**Peel Weight**	0.880 ***	0.830 ***	0.000 ***	0.815 ***	**1**	*2.65* 0.006	*15.27* 0.158	*14.45* 0.552	*18.08* 0.619	*0.35* 0.009
**Juice Content**	−0.114 ***	0.042 ***	0.001 ***	0.112 ***	–0.075 ***	**1**	*27.33* 0.129	*24.29* 0.242	*34.11* 0.360	*5.52* 0.186
**Fruit Weight/Peel Weight**	−0.166 ***	0.020 ***	0.175 ***	0.185 ***	–0.397 ***	0.359 ***	**1**	*2.70* 0.003	*26.13* 0.220	*38.63* 0.056
**Fruit Weight/Juice content**	0.838 n.s.	0.799 n.s.	0.057 ***	0.787 ***	0.743 ***	–0.492 ***	–0.054 ***	**1**	*16.53* 0.682	*34.16* 0.071
**Juice content/Peel Weight**	−0.766 ***	–0.653 ***	0.032 ***	–0.587 ***	–0.787 ***	0.600 ***	0.469 ***	–0.826 ***	**1**	*46.66* 0.081
**Turbidity**	0.022 ***	0.093 ***	0.185 ***	0.010 ***	–0.093 n.s.	0.431 ***	0.236 ***	–0.266 ***	0.284 ***	**1**

**Table 3:** Biometrics of bergamot fruit. Results are presented as the mean value ± standard deviation, n = 8, (2016–2017 and 2017–2018 harvest years). *** significance at *p <* 0.001; ** significance at *p <* 0.01; * significance at *p <* 0.05. Means in the same line are distinguished by small letters. Means in the same column are distinguished by capital letters.

	Cultivar	October	November	December	January	February	March	Sign.
**Fruit Weight** **(g)**	Castagnaro	245 ± 6 eA	277 ± 8 dA	354 ± 14 cA	363 ± 5 cA	397 ± 5 bA	421 ± 7 aA	***
	Fantastico	173 ± 3 dB	201 ± 2 cB	241 ± 4 bB	194 ± 5 cB	266 ± 3 aB	258 ± 12 aB	***
	Femminello	150 ± 2 cC	174 ± 1 bC	131 ± 2 dC	170 ± 2 bC	185 ± 4 aC	146 ± 7 cC	***
	Sign.	***	***	***	***	***	***	
**Peel Weight** **(g)**	Castagnaro	44.01 ± 0.26 cdA	49.77 ± 0.15 bcA	55.01 ± 0.25 bA	55.03 ± 0.67 bA	74.02 ± 5.29 aA	41.97 ± 0.65 dA	***
	Fantastico	31.43 ± 0.21 dB	34.81 ± 0.08 cB	41.13 ± 0.86 bB	42.23 ± 0.67 bB	51.58 ± 0.10 aB	26.53 ± 0.12 eB	***
	Femminello	25.83 ± 0.12 bC	22.8 ± 0.17 cC	20.97 ± 0.06 dC	20.87 ± 0.15 dC	26.90 ± 0.10 aC	19.53 ± 0.50 eC	***
	Sign.	***	***	***	***	***	***	
**Juice** **Content (%)**	Castagnaro	19.73 ± 0.68 fC	21.73 ± 0.21 eB	28.77 ± 0.16 dC	40.08 ± 0.16 aA	36.07 ± 0.49 bB	34.73 ± 0.17 cB	***
	Fantastico	29.33 ± 0.25 eA	30.50 ± 0.36 dA	31.93 ± 0.23 cB	33.57 ± 0.21 bB	30.12 ± 0.03 dC	39.97 ± 0.42 aA	***
	Femminello	21.00 ± 0.10 eB	22.13 ± 0.15 dB	39.03 ± 0.42 bA	33.73 ± 0.21 cB	39.07 ± 0.12 bA	40.01 ± 0.46 aA	***
	Sign.	***	***	***	**	***	***	
**Fruit Weight/Peel Weight**	Castagnaro	5.58 ± 0.13 cAB	5.57 ± 0.18 cB	6.44 ± 0.22 bA	6.60 ± 0.07 bA	5.38 ± 0.32 cB	10.04 ± 0.26 aB	***
	Fantastico	5.50 ± 0.09 aB	5.78 ± 0.06 aB	5.85 ± 0.13 aB	4.59 ± 0.14 cC	5.16 ± 0.05 bB	9.73 ± 0.51 aA	***
	Femminello	5.81 ± 0.05 dA	7.64 ± 0.07 dA	6.26 ± 0.08 cA	8.16 ± 0.08 bB	6.87 ± 0.17 cdA	7.49 ± 0.32 aA	***
	Sign.	*	***	**	***	***	***	
**Fruit Weight/Juice Content**	Castagnaro	12.44 ± 0.17 bcA	12.76 ± 0.29 bA	12.32 ± 0.52 bA	9.07 ± 0.14 dA	11.01 ± 0.25 cdA	12.13 ± 0.15 aA	***
	Fantastico	5.90 ± 0.14 dC	6.60 ± 0.15 cC	7.54 ± 0.15 bB	5.78 ± 0.18 dB	8.83 ± 0.10 aB	6.46 ± 0.31 cB	***
	Femminello	7.14 ± 0.10 bB	7.87 ± 0.04 cB	3.37 ± 0.09 dC	5.05 ± 0.10 dC	4.73 ± 0.12 eC	3.66 ± 0.18 aC	
	Sign.	***	***	***	***	***	***	
**Juice Content/Peel Weight**	Castagnaro	0.45 ± 0.02 eC	0.44 ± 0.01 bC	0.52 ± 0.00 dC	0.73 ± 0.01 aC	0.49 ± 0.04 cC	0.83 ± 0.02 bC	***
	Fantastico	0.93 ± 0.01 bA	0.88 ± 0.01 aB	0.78 ± 0.01 eB	0.79 ± 0.01 cB	0.58 ± 0.00 dB	1.51 ± 0.02 eB	***
	Femminello	0.81 ± 0.01 fB	0.97 ± 0.00 eA	1.86 ± 0.02 bA	1.62 ± 0.02 cA	1.45 ± 0.00 dA	2.05 ± 0.03 aA	***
	Sign.	***	***	***	***	***	***	

**Table 4:** Physico-chemical properties of bergamot juice. Results are presented as the mean value ± standard deviation, n = 8, (2016–2017 and 2017–2018 harvest years). *** significance at *p <* 0.001; ** significance at *p <* 0.01; * significance at *p <* 0.05; n.s., not significant. Means in the same line are distinguished by small letters. Means in the same column are distinguished by capital letters.

	Cultivar	October	November	December	January	February	March	Sign.
**Turbidity** **(%)**	Castagnaro	33.67 ± 0.25 eB	36.55 ± 0.13 cA	34.48 ± 0.28 dB	48.78 ± 0.30 aB	36.37 ± 0.15 cB	39.50 ± 0.17 bB	***
	Fantastico	34.20 ± 0.10 eB	32.28 ± 0.16 fC	36.14 ± 0.09 cA	49.12 ± 0.08 aB	35.27 ± 0.06 dC	39.23 ± 0.21 bB	***
	Femminello	35.27 ± 0.31 dA	35.33 ± 0.23 dB	35.7 ± 0.46 dA	54.03 ± 0.45 aA	38.50 ± 0.26 cA	40.03 ± 0.15 bA	***
	Sign.	**	n.s.	*	*	n.s.	n.s.	
**pH**	Castagnaro	2.4 ± 0.06 cA	2.4 ± 0.06 cA	2.5 ± 0.06 bcA	2.7 ± 0.06 abA	2.7 ± 0.06 abA	2.8 ± 0.06 aA	***
	Fantastico	2.4 ± 0.0 bA	2.4 ± 0.06 bA	2.5 ± 0.10 abAB	2.5 ± 0.10 abAB	2.6 ± 0.12 abA	2.7 ± 0.10 aA	**
	Femminello	2.2 ± 0.12 dB	2.3 ± 0.06 dA	2.3 ± 0.06 dA	2.4 ± 0.06 bcB	2.6 ± 0.0 abA	2.7 ± 0.0 aA	***
	Sign.	**	n.s.	*	*	n.s.	n.s.	
**°Brix**	Castagnaro	9.4 ± 0.06 aA	9.5 ± 0.06 aB	8.3 ± 0.06 dB	8.7 ± 0.10 bB	8.5 ± 0.06 cA	8.3 ± 0.06 cdA	***
	Fantastico	9.1 ± 0.06 bB	10.0 ± 0.10 aA	8.7 ± 0.10 cA	9.1 ± 0.10 bA	8.2 ± 0.20 dAB	7.9 ± 0.60 eB	***
	Femminello	9.1 ± 0.12 aB	8.6 ± 0.06 bC	8.6 ± 0.12 bA	9.3 ± 0.06 aA	8.0 ± 0.06 cB	8.2 ± 0.06 cA	***
	Sign.	**	***	**	***	*	***	
**Titratable Acidity (TA) (g/L)**	Castagnaro	53.86 ± 0.29 aB	51.77 ± 0.06 bB	49.74 ± 0.26 cA	42.2 ± 0.26 dC	40.67 ± 1.42 dC	34.98 ± 0.2 eC	***
	Fantastico	58.67 ± 0.06 bA	59.50 ± 0.10 aA	47.58 ± 0.06 cB	46.31 ± 0.06 dB	46.23 ± 0.13 dB	39.83 ± 0.15 eB	***
	Femminello	54.28 ± 0.32 aB	49.87 ± 0.85 bC	46.63 ± 0.81 cB	55.37 ± 0.32 aA	49.0 ± 0.36 bA	41.90 ± 0.25 dA	***
	Sign.	***	***	***	***	***	***	
**°Brix/TA (%)** **(Maturity Index)**	Castagnaro	1.75 ± 0.02 bcB	1.84 ± 0.01 bcB	1.66 ± 0.01 bAB	2.06 ± 0.02 bB	2.08 ± 0.07 cB	2.38 ± 0.01 aA	***
	Fantastico	1.56 ± 0.01 bcB	1.68 ± 0.02 bB	1.83 ± 0.02 bB	1.97 ± 0.02 dC	1.77 ± 0.05 cdB	1.97 ± 0.02 aA	***
	Femminello	1.68 ± 0.03 bA	1.72 ± 0.02 aA	1.84 ± 0.01 bA	1.67 ± 0.00 aA	1.64 ± 0.00 bA	1.97 ± 0.02 bB	***
	Sign.	**	***	*	***	***	***	
**Formol Number** **(mL NaOH 1N/100mL)**	Castagnaro	2.07 ± 0.06 aA	1.87 ± 0.06 bB	1.43 ± 0.06 cC	2.1 ± 0.10 aA	2.13 ± 0.06 aA	2.03 ± 0.06 abB	***
	Fantastico	2.07 ± 0.06 aA	2.10 ± 0.10 aA	1.97 ± 0.12 aA	1.90 ± 0.10 aA	1.53 ± 0.06 bB	1.60 ± 0.10 bC	***
	Femminello	2.03 ± 0.15 bA	1.47 ± 0.06 cdC	1.67 ± 0.06 cB	1.47 ± 0.06 cdB	1.43 ± 0.06 dB	2.37 ± 0.06 aA	***
	Sign.	n.s.	***	***	***	***	***	
**Vitamin C** **(mg/L)**	Castagnaro	831 ± 7 aB	593 ± 7 cB	672 ± 13 bA	474 ± 12 dC	498 ± 6 dA	341 ± 4 eB	***
	Fantastico	867 ± 6 aA	582 ± 7 bB	566 ± 3 bB	571 ± 9 bA	504 ± 4 cA	457 ± 5 dA	***
	Femminello	669 ± 4 aC	635 ± 7 bA	556 ± 13 cB	543 ± 6 cB	492 ± 4 dA	349 ± 9 eB	***
	Sign.	***	***	***	***	n.s.	***	

**Table 5:** The correlation matrix of the physico-chemical properties of bergamot juice and bergamot cloudy juice which is built on the basis of 48 values for each parameter (4 replicates × 2 harvest years × 3 cultivars). In the south-west section of the matrix is the r-value (above) and the significance level (below) with *p* < 0.05, *. In the north-east section of the matrix there is the *t*-value (in italics) with the significance of the *t*-test calculated at 95% confidence interval and the R^2^ value (underlined).

		pH Juice	°Brix Juice	TA Juice	°Brix/TA/% Juice	Vitamin C Juice	Formol N Juice	Neoeriocitrin Juice	Naringin Juice	Neohesperidin Juice	Melitidin Juice	Brutieridin Juice	Tot. Floids Juice	Neoeriocitrin *Cloudy Juice*	Naringin Cloudy Juice	Neohesperidin Cloudy Juice	Melitidin Cloudy Juice	Brutieridin Cloudy Juice	Tot. Floids Cloudy Juice	DPPH Assay Juice	FRAP Assay Juice
**Juice**	pH	**1**	*76.65* 0.326	*50.76* 0.548	*32.83* 0.169	*31.32* 0.413	*14.31* 0.015	*26.24* 0.120	*36.48* 0.251	*24.92* 0.023	*17.65* 0.146	*21.45* 0.285	*17.55* 0.249	*28.87* 0.378	*53.78* 0.048	*34.16* 0.149	*10.13* 0.046	*24.26* 0.047	*28.44* 0.072	*50.78* 0.060	*50.94* 0.330
	°Brix	−0.571 ***	**1**	*43.67* 0.529	*24.10* 0.153	*30.98* 0.413	*78.41* 0.119	*12.73* 0.309	*28.34* 0.036	*16.67* 0.002	*0.40* 0.072	*15.18* 0.094	*17.30* 0.490	*19.42* 0.184	*43.33* 0.002	*25.30* 0.011	*16.34* 0.180	*8.36* 0.008	*28.41* 0.021	*49.98* 0.272	*41.07* 0.194
	TA	−0.740 ***	0.727 ***	**1**	*18.93* 0.432	*28.73* 0.588	*51.46* 0.010	*32.97* 0.317	*14.50* 0.309	*22.54* 0.001	*40.53* 0.078	*17.97* 0.377	*15.75* 0.147	*23.51* 0.446	*11.54* 0.003	*18.49* 0.177	*46.73* 0.135	*40.02* 0.016	*28.24* 0.025	*44.67* 0.006	*11.34* 0.453
	°Brix/TA (%)	0.411 ***	−0.391 ***	−0.657 ***	**1**	*29.97* 0.008	*33.71* 0.076	*13.39* 0.003	*4.34* 0.046	*4.54* 0.306	*21.51* 0.252	*1.88* 0.003	*16.61* 0.173	*4.55* 0.006	*9.78* 0.278	*0.62* 0.432	*28.29* 0.209	*20.18* 0.004	*25.07* 0.846	*47.54* 0.017	*9.55* 0.109
	Vitamin C	−0.643 ***	−0.643 ***	0.767 ***	0.089 ***	**1**	*31.62* 0.002	*30.63* 0.280	*29.71* 0.392	*30.23* 0.028	*30.96* 0.168	*30.08* 0.513	*3.58* 0.236	*30.22* 0.415	*29.46* 0.002	*29.93* 0.166	*31.19* 0.124	*30.85* 0.016	*25.98* 0.013	*8.27* 0.000	*29.45* 0.926
	FormolNumber	0.124 ***	0.345 ***	−0.098 ***	0.276 ***	−0.042 ***	**1**	*27.60* 0.001	*37.30* 0.161	*25.77* 0.039	*19.40* 0.000	*22.10* 0.030	*17.57* 0.064	*29.82* 0.040	*54.78* 0.078	*35.06* 0.208	*12.91* 0.001	*36.64* 0.001	*28.44* 0.055	*50.86* 0.048	*51.90* 0.002
	Neoeriocitrin	0.346 ***	−0.556 ***	−0.563 ***	0.054 ***	−0.529 ***	0.032 ***	**1**	*17.80* 0.020	*7.56* 0.150	*9.96* 0.004	*8.35* 0.096	*17.07* 0.166	*8.65* 0.303	*26.99* 0.002	*14.26* 0.232	*19.67* 0.003	*7.38* 0.002	*28.39* 0.016	*49.13* 0.014	*26.06* 0.196
	Naringin	0.501 ***	−0.190 ***	−0.556 ***	0.214 ***	−0.626 ***	0.401 ***	0.141 ***	**1**	*8.62* 0.032	*25.67* 0.080	*5.47* 0.759	*16.43* 0.019	*8.67* 0.202	*4.71* 0.100	*3.77* 0.201	*32.18* 0.121	*24.57* 0.162	*28.32* 0.005	*46.93* 0.023	*4.61* 0.390
	Neohesperidin	0.153 ***	0.045 ***	−0.024 ***	−0.553 ***	−0.166 ***	−0.197 ***	−0.387 ***	0.179 ***	**1**	*14.97* 0.599	*1.95* 0.191	*16.80* 0.042	*0.29* 0.006	*14.36* 0.218	*5.17* 0.316	*20.99* 0.100	*13.30* 0.002	*28.36* 0.194	*48.13* 0.022	*14.05* 0.039
	Melitidin	−0.382 ***	0.269 n.s.	0.280 ***	0.502 ***	0.410 ***	0.000 ***	0.066 ***	−0.282 ***	−0.774 ***	**1**	*14.19* 0.174	*17.30* 0.163	*17.01* 0.032	*37.46* 0.077	*22.54* 0.085	*9.54* 0.339	*4.57* 0.008	*28.41* 0.182	*49.91* 0.045	*35.96* 0.161
	Brutieridin	−0.534 ***	0.306 ***	0.614 ***	0.050 n.s.	0.716 ***	−0.173 ***	−0.310 ***	−0.871 ***	−0.437 n.s.	0.417 ***	**1**	*16.69* 0.097	*1.80* 0.236	*9.88* 0.001	*2.40* 0.063	*18.67* 0.104	*12.72* 0.047	*28.35* 0.004	*47.66* 0.001	*9.73* 0.488
	Total Floids	0.499 ***	−0.700 ***	−0.384 ***	−0.416 ***	−0.486 ***	−0.252 ***	0.407 ***	0.137 ***	0.205 ***	−0.404 ***	−0.311 ***	**1**	*16.79* 0.236	*16.25* 0.111	*16.58* 0.010	*17.46* 0.126	*17.22* 0.016	*26.37* 0.125	*11.85* 0.583	*16.25* 0.242
**Cloudy Juice**	Neoeriocitrin	0.615 ***	−0.429 ***	−0.668 ***	0.080 ***	−0.644 ***	0.200 ***	0.550 ***	0.449 ***	−0.077 n.s.	−0.178 ***	−0.486 n.s.	0.486 ***	**1**	*15.17* 0.071	*5.23* 0.370	*24.07* 0.195	*15.37* 0.013	*28.36* 0.026	*48.14* 0.021	*14.78* 0.435
	Naringin	−0.219 ***	−0.048 ***	0.054 ***	0.527 ***	0.048 ***	0.280 ***	−0.040 ***	0.316 ***	−0.467 ***	0.278 ***	0.035 ***	−0.333 ***	−0.266 ***	**1**	*9.19* 0.187	*46.96* 0.031	*37.38* 0.497	*28.30* 0.045	*46.42* 0.068	*0.00* 0.003
	Neohesperidin	−0.386 ***	0.105 ***	0.421 ***	−0.657 n.s.	0.408 ***	−0.456 ***	−0.482 ***	−0.448 ***	0.562 ***	−0.291 ***	0.251 ***	−0.099 ***	−0.608 ***	−0.432 ***	**1**	*29.49* 0.038	*21.27* 0.001	*28.33* 0.358	*47.47* 0.000	*8.97* 0.148
	Melitidin	−0.214 ***	0.424 ***	0.367 ***	0.457 ***	0.352 ***	0.032 ***	−0.052 ***	−0.348 ***	−0.316 ***	0.582 ***	0.323 ***	−0.355 ***	−0.442 ***	0.175 ***	−0.195 ***	**1**	*18.21* 0.171	*28.43* 0.282	*50.46* 0.002	*44.75* 0.165
	Brutieridin	0.217 ***	0.091 ***	0.125 ***	−0.062 ***	0.127 ***	0.022 ***	0.044 ***	−0.403 ***	0.047 ***	0.092 ***	0.217 ***	0.128 ***	0.116 ***	−0.705 ***	0.032 ***	0.414 ***	**1**	*28.40* 0.073	*49.69* 0.065	*35.65* 0.029
	Total Floids	−0.268 ***	−0.146 ***	0.158 ***	−0.920 ***	−0.114 ***	−0.235 ***	−0.126 ***	−0.071 ***	0.441 ***	−0.427 ***	−0.066 ***	0.353 ***	−0.161 ***	−0.213 ***	0.598 ***	−0.531 ***	−0.271 ***	**1**	*26.73* 0.040	*28.30* 0.043
**Juice**	DPPH assay	0.245 ***	−0.522 ***	−0.079 ***	−0.128 ***	0.000 ***	−0.220 ***	0.116 ***	−0.151 ***	0.149 ***	0.211 ***	0.024 ***	0.764 ***	0.143 ***	−0.260 ***	0.017 ***	−0.048 ***	0.256 ***	0.199 ***	**1**	*46.40* 0.001
	FRAP assay	−0.574 ***	0.441 ***	0.673 ***	−0.330 ***	0.962 ***	0.044 ***	−0.309 ***	−0.624 ***	−0.196 ***	0.402 ***	0.699 ***	−0.492 ***	−0.659 ***	0.054 n.s.	0.385 ***	0.406 ***	0.171 ***	−0.206 ***	0.024 ***	**1**

**Table 6:** Main flavonoids in bergamot juice. Results are presented as the mean value ± standard deviation, n = 8, (2016–2017 and 2017–2018 harvest years). *** significance at *p <* 0.001; ** significance at *p <* 0.01; * significance at *p <* 0.05. Means in the same line are distinguished by small letters. Means in the same column are distinguished by capital letters.

	Cultivar	October	November	December	January	February	March	Sign.
**Neoeriocitrin** **(%)**	Castagnaro	10.16 ± 0.08 eC	13.85 ± 0.11 cB	10.14 ± 0.05 eC	12.64 ± 0.17 dA	20.58 ± 0.19 aB	17.95 ± 0.12 bA	***
	Fantastico	12.84 ± 0.07 cB	12.35 ± 0.05 dC	12.82 ± 0.08 cB	11.72 ± 0.09 eB	22.75 ± 0.05 aA	16.67 ± 0.06 bC	***
	Femminello	15.26 ± 0.19 cA	15.98 ± 0.29 bA	15.35 ± 0.11 cA	10.30 ± 0.05 dC	15.23 ± 0.03 cC	17.41 ± 0.15 aB	***
	Sign.	***	***	***	***	***	***	
**Naringin** **(%)**	Castagnaro	29.13 ± 0.06 dB	28.62 ± 0.26 dA	29.01 ± 0.15 dB	39.52 ± 0.47 bA	33.36 ± 0.18 cA	42.61 ± 0.13 aA	***
	Fantastico	25.15 ± 0.08 dC	26.35 ± 0.05 cB	26.11 ± 0.06 cC	28.42 ± 0.09 aC	27.21 ± 0.16 bB	28.63 ± 0.12 aB	***
	Femminello	30.69 ± 0.19 cA	23.92 ± 0.20 eC	30.8 ± 0.05 cA	37.18 ± 0.03 bB	26.65 ± 0.03 dC	42.3 ± 0.26 aA	***
	Sign.	***	***	***	***	***	***	
**Neohesperidin** **(%)**	Castagnaro	17.87 ± 0.06 bC	16.75 ± 0.20 cC	17.75 ± 0.13 bB	33.11 ± 0.10 aA	13.82 ± 0.12 dC	17.92 ± 0.07 bC	***
	Fantastico	21.05 ± 0.23 bA	20.51 ± 0.12 cA	20.06 ± 0.23 cA	29.16 ± 0.08 aC	18.51 ± 0.11 dB	29.34 ± 0.24 aA	***
	Femminello	18.35 ± 0.18 eB	19.51 ± 0.17 cB	18.01 ± 0.10 eB	32.62 ± 0.15 aB	22.91 ± 0.02 bA	18.78 ± 0.03 dB	***
	Sign.	***	***	***	***	***	***	
**Melitidin** **(%)**	Castagnaro	11.65 ± 0.29 bA	13.32 ± 0.21 aA	11.51 ± 0.13 bA	5.21 ± 0.08 dC	13.68 ± 0.11 aA	7.13 ± 0.11 cA	***
	Fantastico	8.13 ± 0.09 cB	8.36 ± 0.08 bC	8.19 ± 0.10 b cC	7.23 ± 0.06 dA	9.23 ± 0.07 aB	3.30 ± 0.10 eB	***
	Femminello	10.56 ± 0.06 aB	10.43 ± 0.15 aB	10.51 ± 0.03 aB	6.91 ± 0.03 dB	8.27 ± 0.03 bC	7.22 ± 0.08 cA	***
	Sign.	***	***	***	***	***	***	
**Brutieridin** **(%)**	Castagnaro	31.49 ± 0.05 aB	27.46 ± 0.12 bC	31.47 ± 0.15 aB	9.52 ± 0.08 eC	18.56 ± 0.07 cC	14.39 ± 0.16 dB	***
	Fantastico	32.83 ± 0.07 aA	32.43 ± 0.16 bA	32.82 ± 0.08 aA	23.47 ± 0.16 cA	22.3 ± 0.13 dB	22.06 ± 0.07 dA	***
	Femminello	25.50 ± 0.11 cC	30.16 ± 0.05 aB	25.33 ± 0.05 cC	12.99 ± 0.12 eB	26.94 ± 0.14 bA	14.29 ± 0.01 dB	***
	Sign.	***	***	***	***	***	***	
**Total Flavonoids** **in juice (g/L)**	Castagnaro	276 ± 3 dC	212 ± 7 eC	488 ± 11 aA	425 ± 7 bB	419 ± 8 bC	348 ± 18 cC	***
	Fantastico	361 ± 9 cA	303 ± 6 dA	233 ± 17 eC	509 ± 8 bA	675 ± 10 aB	678 ± 4 aB	***
	Femminello	287 ± 1 eB	408 ± 4 cB	364 ± 4 dB	390 ± 12 cC	845 ± 11 aA	824 ± 7 bA	***
	Sign.	***	***	***	***	***	***	

**Table 7:** Main flavonoids in the aqueous extract: cloudy juice. Results are presented as the mean value ± standard deviation, n = 8, (2016–2017 and 2017–2018 harvest years). *** significance at *p <* 0.001; ** significance at *p <* 0.01; * significance at *p <* 0.05. Means in the same line are distinguished by small letters. Means in the same column are distinguished by capital letters.

	Cultivar	October	November	December	January	February	March	Sign.
**Neoeriocitrin** **(%)**	Castagnaro	17.74 ± 0.09 dB	25.53 ± 0.10 bA	17.86 ± 0.11 dA	18.66 ± 0.09 cC	25.23 ± 0.15 bA	28.89 ± 0.14 aB	***
	Fantastico	18.06 ± 0.10 dA	16.43 ± 0.09 eC	18.09 ± 0.14 dA	25.74 ± 0.07 bA	24.3 ± 0.20 cB	26.13 ± 0.17 aC	***
	Femminello	16.23 ± 0.12 eC	23.36 ± 0.23 bB	16.33 ± 0.07 eB	19.11 ± 0.10 dB	20.16 ± 0.18 cC	33.49 ± 0.05 aA	***
	***	***	***	***	***	***	***	
**Naringin** **(%)**	Castagnaro	39.52 ± 0.18 bB	35.78 ± 0.18 dB	39.35 ± 0.09 bB	37.64 ± 0.08 cA	31.35 ± 0.10 eC	42.07 ± 0.15 aA	***
	Fantastico	33.44 ± 0.23 cC	38.98 ± 0.11 aA	33.50 ± 0.10 cC	27.18 ± 0.16 eC	34.38 ± 0.08 bA	29.12 ± 0.25 dC	***
	Femminello	42.20 ± 0.11 aA	33.36 ± 0.07 cC	42.33 ± 0.06 aA	31.17 ± 0.06 eB	32.31 ± 0.04 dB	37.96 ± 0.06 bB	***
	***	***	***	***	***	***	***	
**Neohesperidin** **(%)**	Castagnaro	25.71 ± 0.27 bC	20.76 ± 0.12 dC	25.64 ± 0.2 bC	27.08 ± 0.06 aC	22.05 ± 0.22 cC	18.91 ± 0.15 eB	***
	Fantastico	32.29 ± 0.25 aA	24.26 ± 0.12 dB	32.15 ± 0.07 aA	31.19 ± 0.16 bB	22.69 ± 0.13 eB	30.43 ± 0.15 cA	***
	Femminello	29.52 ± 0.34 cB	28.25 ± 0.28 dA	29.22 ± 0.03 cB	35.73 ± 0.09 aA	32.19 ± 0.03 bA	16.11 ± 0.09 eC	***
	***	***	***	***	***	***	***	
**Melitidin** **(%)**	Castagnaro	6.22 ± 0.09 cA	6.52 ± 0.06 bB	6.24 ± 0.12 b cA	6.24 ± 0.08 b cA	7.04 ± 0.18 aA	2.60 ± 0.05 dA	***
	Fantastico	4.28 ± 0.07 cB	7.81 ± 0.15 aA	4.27 ± 0.10 cC	3.82 ± 0.15 dB	6.69 ± 0.14 bB	2.38 ± 0.08 eB	***
	Femminello	4.45 ± 0.14 aB	4.3 ± 0.24 aC	4.55 ± 0.05 aB	3.37 ± 0.07 bB	4.32 ± 0.07 aC	2.13 ± 0.05 cC	***
	***	***	***	***	***	***	***	
**Brutieridin** **(%)**	Castagnaro	10.81 ± 0.07 cB	11.61 ± 0.23 bB	10.91 ± 0.14 cB	10.38 ± 0.14 dB	14.33 ± 0.08 aA	7.53 ± 0.10 eC	***
	Fantastico	11.93 ± 0.15 bA	12.52 ± 0.15 aA	11.99 ± 0.06 bA	12.07 ± 0.16 bA	11.94 ± 0.15 bB	11.94 ± 0.24 bA	**
	Femminello	7.56 ± 0.32 cC	10.73 ± 0.22 a bC	7.56 ± 0.06 cC	10.62 ± 0.08 abB	11.02 ± 0.06 aC	10.31 ± 0.07 bB	***
	***	***	***	***	***	***	***	
**Total Flavonoids** **in Cloudy Juice (mg/L)**	Castagnaro	4272 ± 17 cC	4027 ± 12 dC	4778 ± 22 bC	6546 ± 34 aC	4066 ± 14 dC	4273 ± 18 cC	***
	Fantastico	6680 ± 28 cB	5926 ± 12 dB	7816 ± 14 bB	7960 ± 25 aB	5671 ± 30 eB	6671 ± 34 cB	***
	Femminello	8271 ± 82 dA	7753 ± 42 eA	8489 ± 12 cA	8651 ± 9 bA	9254 ± 10 aA	8212 ± 23 dA	***
	***	***	***	***	***	***	***	

**Table 8:** Antioxidant activity of bergamot juice. AAE = ascorbic acid equivalent. Results are presented as the mean value ± standard deviation, n = 8, (2016–2017 and 2017–2018 harvest years). *** significance at *p <* 0.001; ** significance at *p <* 0.01; * significance at *p <* 0.05. Means in the same line are distinguished by small letters. Means in the same column are distinguished by capital letters.

	Cultivar	October	November	December	January	February	March	Sign.
**DPPH Assay—Juice** **(mg AAE/100mL)**	Castagnaro	380.8 ± 1.16dC	320.3 ± 2.30eC	494.9 ± 2.17aA	431.2 ± 1.11bA	386.6 ± 2.21cC	305.6 ± 1.85fC	***
	Fantastico	423.4 ± 2.17bA	361.7 ± 3.79cB	340.7 ± 1.70dB	416.8 ± 2.41bB	453.3 ± 4.62aB	450.9 ± 4.23aB	***
	Femminello	394.3 ± 4.01cB	420.6 ± 5.11bA	330.8 ± 3.53dC	340.0 ± 7.71dC	478.4 ± 6.59aA	473.9 ± 4.10aA	***
	Sign.	***	***	***	***	***	***	
**FRAP Assay—Juice** **(mM AAE 100/mL)** **Formol Number** **(mL NaOH 0.1N/100mL)**	*Castagnaro*	45.21 ± 0.96aA	34.44 ± 1.93cdB	40.67 ± 0.56bA	32.66 ± 0.10dB	35.77 ± 0.47cA	28.41 ± 0.08eB	***
	*Fantastico*	45.84 ± 0.39aA	36.93 ± 0.64bAB	35.91 ± 0.64bcB	35.05 ± 0.64cdA	34.17 ± 0.75dA	33.65 ± 0.57dA	***
	*Femminello*	39.67 ± 0.28aB	38.04 ± 0.58aA	35.24 ± 0.66bB	33.89 ± 0.78AbcB	31.82 ± 0.76cB	26.26 ± 1.35dC	***
	Sign.	***	*	***	**	***	***	
